# Current Situation, Determinants, and Solutions to Drug Shortages in Shaanxi Province, China: A Qualitative Study

**DOI:** 10.1371/journal.pone.0165183

**Published:** 2016-10-25

**Authors:** Caijun Yang, Lina Wu, Wenfang Cai, Wenwen Zhu, Qian Shen, Zongjie Li, Yu Fang

**Affiliations:** 1 Department of Pharmacy Administration and Clinical Pharmacy, School of Pharmacy, Xi'an Jiaotong University, Xi’an, China; 2 Center for Drug Safety and Policy Research, Xi'an Jiaotong University, Xi’an, China; Jagiellonian University, POLAND

## Abstract

**Objective:**

Drug shortages were a complex global problem. The aim of this study was to analyze, characterize, and assess the drug shortages, and identify possible solutions in Shaanxi Province, western China.

**Methods:**

A qualitative methodological approach was conducted during May–June 2015 and December 2015–January 2016. Semi-structured interviews were performed to gather information from representatives of hospital pharmacists, wholesalers, pharmaceutical producers, and local health authorities.

**Results:**

Thirty participants took part in the study. Eight traditional Chinese medicines and 87 types of biologicals and chemicals were reported to be in short supply. Most were essential medicines. Five main determinants of drug shortages were detected: too low prices, too low market demands, Good Manufacturing Practice (GMP) issues, materials issues, and approval issues for imported drugs. Five different solutions were proposed by the participants: 1) let the market decide the drug price; 2) establish an information platform; 3) establish a reserve system; 4) enhance the communication among the three parties in the supply chain; and 5) improve hospital inventory management.

**Conclusions:**

Western China was currently experiencing a serious drug shortage. Numerous reasons for the shortage were identified. Most drug shortages in China were currently because of “too low prices.” To solve this problem, all of the stakeholders, especially the government, needed to participate in managing the drug shortages.

## Background

The World Health Organization (WHO) considered drug shortages to be a complex global challenge [1]. Both developing and developed countries were affected by drug shortage problems [2], which seemed to be worse in recent years. In the US for example, according to statistics from the University of Utah hospitals and clinics, the number of drug shortages increased sharply during the last 10 years and in 2010, an additional 211 new drug shortages were identified [3]. The situation in Europe was also not optimistic. In November 2014, the European Association of Hospital Pharmacists reported that 86% of 607 practitioners in 36 European countries had sourcing problems, and that 66% said these problems arose on a daily or weekly basis [4]. An urgent appeal was made for worldwide actions on drug shortages.

Drug shortages influenced all stakeholders in the supply chain, especially patients and hospitals, which has raised public concerns. Drug shortages for patients can lead to suboptimal care and delays or cancellation of treatment or surgery [5,6]. Patients may also experience medication errors, adverse outcomes, and increased healthcare costs [7]. At the hospital level, considerable time, effort, and personnel were required to respond to drug shortages [8]. Four types of actions were generally taken to manage drug shortages: 1) monitor and move stock to avoid a shortage; 2) anticipate the extent of shortages and identify therapeutic alternatives; 3) change dispensing practice or allocate remaining stocks; and 4) communicate with other organizations (wholesalers, manufacturers, or hospitals) or establish contracts with new suppliers to secure back-up sources of drugs [9]. The economic burden of these efforts was substantial. A 2011 survey in the US estimated labor costs in hospitals associated with drug shortages of $216 million [3]. Wholesalers and producers may experience reduced profit from the lost business. In addition, their reputation and relationship with authorities and clients also may be affected by unsatisfied demands. Governments had to address this problem; the Center for Drug Evaluation and Research in the US Food and Drug Administration (FDA) even arranged a drug shortage program to deal with all the unsatisfied drug supply problems [10–12].

The causes of drug shortages were complex and diverse, and differ among regions and drug categories. The factors contributing to disruptions in the availability of medicines in the US was summarized [9]: 1) disruptions in the supply of raw or bulk materials; 2) manufacturing difficulties and regulatory issues (such as a capacity crunch); 3) voluntary recalls; 4) change in product formulation or manufacturer; 5) manufacturers’ production decisions and economics (such as the drug price was low or the demand was small); 6) industry consolidations; 7) restricted drug production distribution and allocation; 8) inventory management (which may appear at any node of the supply chain, such as manufacturers, wholesalers, or hospitals/retailers); 9) unexpected increases in demand and shifts in clinical practice; 10) gray-market or alternative distributors; and 11) natural disasters. One or more of these factors can cause drug shortages. For example, in China since the beginning of 2015, there has been a shortage of human serum albumin because of disruptions in the supply of raw materials. For some emergency medicines, shortages were caused by the combination of weak demand, low price, and inventory problems. Different factors required different solutions, which made this problem much more complex.

To the best of our knowledge, most studies were mainly on drug shortages in the US, Europe, and Canada. Several researchers exploring drug shortages in the US investigated its scope, causes, impact, and solutions [3,9,13]. Reports about shortages of medicines in Europe concentrated on the scope and causes [14–16]. Recently, drug shortages in Canada were also discussed and the related reasons were analyzed [17–19].

Drug shortages were not a new problem in China. In early 2004, there were continuous drug shortages and some researchers claimed that the retail price controlled by China’s National Development and Reform Commission was a key cause, although its original objective was to control drug price [20]. An empirical study on the relationship between drug shortages and price-lowering policy during 1997–2007 showed that the policy had widened the gap between market demand and the supply of those medicines [21].

In recent years, news and reports on drug shortages were increasing, especially after the implementation of China’s National Essential Medicines Policy (NEMP) in 2009. Key components of the NEMP included: establishing a national essential medicine list (EML) which included 307 generic medicines and was expanded to 520 in 2012, organizing production, quality assurance, pricing, tendering and procurement, distribution, rational use, monitoring and evaluation, etc. Provincial governments were allowed to supplement the EML to meet their local needs. The NEMP mandated that all the primary health-care institutions must acquire and use essential medicines. For secondary and tertiary hospitals, a certain proportion of all medicines used should be on the EML and this number was decided by provincial governments. To achieve the lowest possible procurement prices, provincial governments had to organize public bidding for essential medicines. Therefore, the price of most essential medicines became lower than before. For example, a research conducted in Shaanxi Province found that the median price of 29 generic essential medicines fell, by 5.2% in the public sector and 4.7% in private pharmacies from 2009 to 2011 [22]. At the same time the availability of those essential medicines, which was already low in 2009, further decreased in 2011. Drug shortage, especially shortage of essential medicines, was not just a local problem and some drugs in shortage had serious and wide influences. For instance, in 2011, protamines were in short supply in the whole country, which seriously affected the ability to perform extracorporeal circulation operations for heart diseases [23]. In 2013, some cities could not supply methimazoles for hyperthyroidism [24]. In early 2015, many hospitals reported shortages of some emergency medicines, such as pralidoxime chloride and noradrenaline [25]. Drug shortages became a critical national problem. However, there were limited researches to report the situation in China, and studies of the reasons and solutions for drug shortages were rare.

The objective of this study was to analyze, characterize, and assess the drug shortages in Shaanxi Province, western China. We focused on investigating the reality of the drug shortage problem to identify its determinants and possible solutions. For the purpose of this study, a shortage of medicine was defined as a situation in which the total supply of an approved drug was inadequate to meet the current or projected demand at the user level [26].

## Method

### Study design and sampling

We conducted the study in Shaanxi Province, which was ranked 15th for GDP per capita among all the 31 provinces in mainland China in 2014 [27]. With a population of 37.93 million and 11 areas in its jurisdiction, Shaanxi Province was broadly representative of the typical health and health system status of the 12 western provinces of China. As such, in 2012, the Ministry of Health of China and WHO selected Shaanxi Province as one of three pilot regions for the Western Area Health Initiative, which was implemented from 2012 to 2015 to improve people’s health and ultimately lead to longer life expectancy and a better quality of life in western China [28]. To make sure the supply of medicines, especially the essential medicines, was very important in guaranteeing people’s health.

A qualitative methodological approach was employed to characterize and assess the problem of drug shortages in Shaanxi. The main reason was that most organizations did not keep a track of their data on drug shortages in the supply chain in China, not to mention reporting these data. Consequently, using quantitative methods became tough. Finally, a semi-structured interview was chosen to achieve the study objectives. Information was collected from different stakeholders, including hospital pharmacists (hospital pharmacists were most familiar with drug shortages as 80% of medicines were dispensed in Hospital pharmacies in China [29]), pharmaceutical wholesalers (they were also usually responsible for distributing), pharmaceutical manufacturers, and local health authorities. Interviewees were selected from hospitals, manufacturers, wholesalers, and authorities according to the following principles:

#### Selection of hospital representatives

One research conducted in Shaanxi Province concluded that inadequate governmental funding and inefficient procurement systems might lead to lower availability of medicines in public hospitals [22]. Hospitals located in lower income areas were more easily getting inadequate governmental funding, and they had a weaker incentive to invest in procurement system. Therefore, to make our survey be more representative, we used the income of different areas as the criterion for choosing hospitals. According to the GDP per capita in 2014, the 11 areas in Shaanxi can be classified into three income strata (high, middle, low). We included the highest and lowest income areas in each stratum (Xi’an and Yulin, Xianyang and Baoji, and Shangluo and Weinan, respectively). In all areas except Xi’an, we aimed to include one tertiary hospital in the city and two secondary hospitals located in two different counties. As the provincial capital area, Xi’an had more tertiary hospitals than other areas. We chose three tertiary hospitals and two secondary hospitals in Xi’an for interviews. All hospitals were randomly selected and they were state owned. In each hospital, the interviewee was selected according to the following steps: a) on the day of the interview, if the director of the hospital pharmacy was available, then he/she would be our first choice; b) if the director was not available, then the purchase supervisor or the inventory supervisor was randomly selected; and c) if all three staff members were not available, we did not interview other workers in the hospital to guarantee the quality of information. Another hospital which was in the same grade and located in the same area would be selected to assure the sampling size.

#### Selection of manufacturers and wholesalers

To make the face-to-face interview convenient, we only chose the local manufacturers and wholesalers. The list of production companies granted Good Manufacturing Practice (GMP) certificates and wholesalers granted Good Supply Practice certificates was found on the official website of the Shaanxi Food and Drug Administration. For production companies, we sorted them according to the number of approved drugs they had, from most to least. For one missing medicines (known from the results of the interview with hospital pharmacists implemented previously), if more than one production company supplied it, then the one who had higher prioritization rank was chosen for interview. Finally, five production companies were selected. Similarly, considering the production line and the capacity of each wholesaler, three wholesalers were also chosen. Most of the drugs known to be in short supply were covered by the five production companies and three wholesalers. In each production company, one sales manager was chosen for the interview. At pharmaceutical wholesalers, both sales managers and purchasing managers were familiar with the drug shortage phenomenon. Therefore, either one who was available would be our interviewee. But we only included one interviewee at each wholesaler. In total, there were eight interviewees from five manufacturers and three wholesalers.

#### Selection of authorities

Local authorities who were most familiar with the drug shortage problems were the Bureau of Pharmaceutical Affairs of Shaanxi Health and Family Planning Commission (it was responsible for implementing the national essential medicine system in Shaanxi Province) and the Drug Procurement Center of Shaanxi Food and Drug Administration (it was responsible for centralized pubic bidding in drug purchasing of state-owned medical institutions). Therefore, one upper-level manager from each department was interviewed to represent the voices from provincial government.

During the preparatory phase, topic guidelines (S1–S4 Files) were constructed for the above four groups based on a literature review. The interview guidelines were refined by pre-interviews with representatives from the four groups (one representative in each group). The guidelines contained the following topics: 1) basic information about the interviewees and their organization; 2) present drug shortages in their organization to their knowledge; 3) the causes of drug shortages; and 4) possible solutions for this problem.

### Data collection

The interviews were conducted in stages. From May to June 2015, a total of 20 pharmacists in different hospitals were interviewed. From December 2015 to January 2016, eight managers and two authorities participated in the study. All interviews were carried out by two researchers face to face. During the interview, one researcher asked questions and the other kept notes. By the end of each interview, the researcher keeping notes had to check with the interviewee point by point to make sure of the accuracy of their notes. All interviews took place at the interviewee’s offices and lasted between 45 and 80 min.

### Data analysis

Interviews were audio-recorded, transcribed verbatim, and analyzed by four authors. An initial categorizing system was established by the first and third authors based on the interview guides. Each interview was divided into several segments, and categorized according to the initial categorizing system by the fourth and fifth authors. During this process, the first thematic index was modified and subcategories would be added when they arose. All four authors had regular discussions to check that they had a common understanding of the categories generated. When disagreement occurred, the original transcripts were referenced and suggestions from other authors were also considered to reach an agreement. The final thematic index was generated and all data were coded according to this index.

### Ethics

During pre-research, we found that our interviewees were happy to participate in our study, but that they refused to sign the consent form. One direct reason was that one possible cause of drug shortage was the government policies and they need to provide some comments on that. Our interviewees were very cautious over that. Therefore before the interview, we read the consent form to our interviewee and asked for their permission of audio-recording. The interview began only when they agreed to participate. The Ethics Committee of Xi’an Jiaotong University Health Science Center granted ethical approval for the study and they also approved this consent procedure.

## Results

### Sociodemographic profile of the interviewees

As shown in Table 1, the majority of the 30 interview participants were male (20, 66.7%), with an average of 16.9 years of work experience related to medicine. Nearly half of them were aged between 41 and 50 years, twenty were from hospitals, five from pharmaceutical companies, three from wholesale distributors, and two from local authorities.

**Table 1:** The characteristics of interviewees.

Demographic	Interviewees (n = 30)
Sex (n, %)	
Male	20(66.7)
Female	10(33.3)
Age (n, %)	
<30 years	5 (16.7)
30–40 years	6 (20.0)
41–50 years	13(43.3)
>51 years	6 (20.0)
Medicine related work experience (mean ± SD)	16.9±11.2
Representative of stakeholders (n, %)	
Hospital	20(66.7)
Pharmaceutical company	5(16.7)
Wholesaler	3(10.0)
Local health authority	2(6.6)

### The current situation of drug shortages in different organizations

All 28 participants from hospitals, pharmaceutical companies, and wholesale distributors indicated that their organizations experienced drug shortages during the last year. The two respondents from health authorities also highlighted the seriousness of the phenomenon of drug shortages from their knowledge.

The drug shortage list gathered in the second stage was covered by the hospitals’ list. Therefore, we analyzed the mentioned drugs in short supply in the hospitals. The hospital pharmacists reported 8 traditional Chinese medicines and 87 types of biologicals and chemicals to be in short supply. According to the classification given by the 2012 version of “national essential medicine list” [30], the biologicals and chemicals were arranged into classes (Table 2). There were 22 categories of biologicals and chemicals reported by the 20 hospitals. Most agents involved were antimicrobials and cardiovascular drugs, with nine medicines in each category. It was important to note that there were 51 types of national essential medicines and 8 types of locally selected supplementary medicines. The drug shortage phenomenon also varied in the different hospital levels. Antimicrobials, biologicals, and anticancer drugs were the most common drugs in short supply at tertiary hospitals. There was a shortage of cardiovascular drugs, hormones, and endocrine system agents at secondary hospitals. Overall, there was a shortage of more drugs in secondary hospitals than in tertiary hospitals. Most of the imported drugs in short supply were reported in tertiary hospitals.

**Table 2:** Reported shortages of biologicals and chemicals by the interviewed hospitals.

Category	Agents involved	N	NT (%)	NS (%)
Antimicrobials	Benzylpenicillin*; Cefazolin*; Cefoperazone**; Ceftriaxone*; Cefuroxime*; Isoniazid*; Linezolid injection (I); mupirocin ointment (I); Piperacillin (I)	9	4(44.4)	6(66.7)
Cardiovascular drugs	Deslanoside*; Digoxin*; Adrenaline*; Enalapril*; Isoprenaline*; Isosorbide dinitrate*; Noradrenaline*; K strophanthin; Nifedipine(I)	9	2(22.2)	9(100.0)
Digestive system drugs	Anisodamine*; Atropine*; Belladonna*; Domperidone suspension*; Metoclopramide*; Multienzyme tablets**; Inosine tablets; Scopolamine	8	2(25.0)	6(75.0)
Hormones and Endocrine system agents	Chorionic gonadotrophin*; Dexamethasone*; Progesterone*; Thiamazole*; Diethylstilbestrol**; Levothyroxine sodium**; Insulin (I); Salmon calcitonin injection	8	2(25.0)	8(100.0)
Psychotherapeutic agents	Alprazolam*; Chlorpromazine*; Diazepam*; Oryzanol**; Paroxetine hydrochloride (I); Sertraline hydrochloride (I)	6	2(33.3)	4(66.7)
Biologicals	Snake venom antiserum*; Chymotrypsin*; Human immunoglobulin(PH4); Human gamma globulin; Human serum albumin; Purified protein derivative of BCG	6	4(66.7)	5(83.3)
Anti-cancers	Cyclophosphamide*; Daunorubicin*; Doxorubicin*; Methotrexate*; Bleomycin	5	4(80.0)	1(20.0)
Nervous system agents	Betahistine*; Citicoline sodium*; Nikethamide*; Phenytoin sodium*; Dimefline	5	2(40.0)	3(60.0)
Vitamins/ Mineral drugs	Calcium gluconate*; Vitamin B6*; Vitamin K1/K3*; Compound vitamin B**; Junior chewing vitamin tablets	5	1(20.0)	4(80.0)
Blood system agents	Protamine*; Menadione; Ferrous gluconate; Aspirin (I)	4	1(20.0)	4(80.0)
Respiratory system agents	Budesonide; Compound ipratropium bromide	2	0 (0.0)	2(100)
Gynecologicals	Clotrimazole*; Ethacridine lactate*; Oxytocin*	3	2 (66.7)	2(66.7)
Analgesics/Antipyretic/ Anti-inflammatory/ Anti-rheumatism/ gout suppressant	Allopurinol*; Ibuprofen suspension*; Compound aminophenazone and barbital**	3	1 (33.3)	2 (66.7)
Antidotes	Acetamide*; Pralidoxime Chloride*; Pralidoxime iodide*	3	3(100.0)	1(33.3)
Electrolyte/Acid-base balance	Potassium chloride*; Sodium bicarbonate*	2	1(50.0)	1(50.0)
Anesthetics	Ketamine*; Articaine hydrochloride and epinephrine tartrate injection	2	1(50.0)	2(100)
Ophthalmic reparations	Hyaluronic acid sodium eye drops	1	1(100.0)	0(0.0)
Diagnostic agents	Indocyanine green	1	1(100.0)	0(0.0)
Planned Parenthood	Desogestrel and ethinylestradiol tablets	1	1(100.0)	0(0.0)
Antiparasitic drugs	Albendazole (I)	1	0(0.0)	1(100.0)
Urinary system drugs	Hydrochlorothiazide*	1	0(0.0)	1(100.0)
Dermatologicals	Halcinonide solution	1	1(100.0)	0(0.0)
Total		87	36(41.4)	63(72.4)

*: National essential medicines

**: locally selected supplementary medicines

N: Number of drugs reported shortage by all the hospitals; NT: Number of drugs reported shortage by tertiary hospitals;NS: Number of drugs reported shortage by secondary hospitals.

According to the majority of interviewees, they received drug shortage information from their upstream suppliers and delivered the information to their downstream customers or other staff in the same organization. However, most of the information was communicated only when demanded and none of the participants reported early warnings. In particular, local authorities mentioned only that they communicated the notification from the state government, such as designated production information for certain drugs in shortage. A shortage information-reporting channel for public hospitals was available in the information system of the Drug Procurement Center. However, most hospitals did not use it and hospitals could not see the reports from other hospitals.

Interviewees from hospitals and companies had different attitudes towards dealing with drug shortages. When the hospitals placed an order, the wholesalers would satisfy their demands as soon as possible. If the hospitals cannot get the medicines from their wholesalers in time, their strategy would be to wait or call more frequently to urge their original wholesalers to act. When some medicines were out of stock, physicians would prescribe substitutive medications or tell the patients to buy them from somewhere else.

Most producers showed their incapability to cope with drug shortages and said they could not do anything if the drug price was too low or the material was too expensive or insufficient. All producers made their production plans according to the previous year’s sales or those of several years before. When there was more unexpected demand, producers could sometimes ramp up manufacturing in response. More typically, it was too late for producers to boost their production because the production lead time was always very long.

However, wholesalers had more positive coping methods. They usually had two other strategies other than urging the production companies to meet the demand. The first strategy was to order drugs in shortage from other wholesalers who had inventories to meet the hospitals’ demand, but with a higher cost. The second strategy was to find other production companies who could produce and deliver these drugs in time. However, it was always the last choice for wholesalers because switching to another manufacturer who can produce and deliver these drugs requires the approval of hospitals, which means a long processing time. Two wholesalers had prevention measures in place, such as investing in order-processing and information systems and implementing suitable order strategies to eliminate drug shortages caused by ordering errors. One wholesaler even established a performance-evaluation system to encourage their employees to try their best to avoid drug shortages or lower the effect to a minimum level.

### Causes of drug shortages

Drug shortages were a complex multifaceted phenomenon. Interviewees were asked to express their opinion on the causes of drug shortages. A number of reasons were mentioned and the most common ones proposed by different stakeholders were extracted for analysis. According to the frequency given by interviewees, the most important common reasons were ranked in Table 3 in a descending order.

**Table 3:** Causes of drug shortages proposed by different stakeholders.

Causes of drug shortages	Representative drugs
1) Too low prices	Dexamethasone; Thiamazole; Deslanoside; Adrenaline; Alprazolam.
2) Too low market demands	Pralidoxime Chloride; Pralidoxime Iodide; Snake venom antiserum; Ketamine.
3) GMP issues	Doxorubicin; Cefuroxime; Hyaluronic acid sodium eye drops.
4) Materials issues	Human serum albumin; Human gamma globulin; Clotrimazole.
5) Approval problems of imported drugs	Mupirocin ointment; Linezolid Injection; Piperacillin; Paroxetine Hydrochloride.

Nearly all of the respondents believed that too low prices were the causes of shortages of most drugs. Competitive public bidding was the most common way for manufacturers to reach China’s market and usually only manufacturers who provided the lowest price can win the bid once they meet the quality requirement. The bid-winning price plus a government-approved retail price markup may not exceed the allowed guidance retail price which was set by the National Development and Reform Commission (NDRC). Since 1997, the central authority reduced drug prices more than 30 times. Competitive public bidding and a compulsory price reduction set the prices for lots of medicines at an unprofitable level for manufacturers. Although the Chinese Government announced to lift price controls on most medicines since June 1, 2015, the participants from production companies and wholesalers were still concerned. The public bidding was the biggest challenge as before. Besides, new pressure was coming from the second negotiation which allowed a city or a hospital alliance (several hospitals get together) to negotiate a new price (lower than the bidding price) with manufacturers. Therefore, it would still be very difficult for manufacturers to sell drugs to hospitals at a “reasonable” price. A too low market demand was the main cause of the shortage of emergency medicines and orphan drugs. Even though every hospital had to prepare a certain amount of emergency medicines, the market requirements were still low. The low prices and short expiry date for most emergency medicines worsened the situation because none of these production companies or wholesalers liked to keep sufficient stock. Orphan drugs also had the same problem of low market demand, especially those for children. A pharmacist from a secondary hospital offered an example: “This year we kept ordering ketamines, but all five wholesalers delivered nothing. The clinical requirement in our hospital is small, even for our county, but it is necessary. However, I know none of the hospitals in this county have ketamines, except for some tertiary hospitals in big cities.”

The third and fourth reasons were related to manufacturing: GMP and materials issues. The GMP issue referred to the renewal of GMP authentication. China Food and Drug Administration was responsible for medicines’ GMP authentication. The validity period for one GMP authentication was five years. The production company had to re-apply for renewal their authentication six months before the expiration. Producers and wholesalers complained about the long process of renewing their product authentication, which usually took half a year or more. One wholesaler said: “During the renewal of our authentication, precise delivery becomes very difficult.” Materials issues included the shortage and the high prices of raw materials. Human serum albumin and human gamma globulin were representative of products affected by shortages of raw materials. The prices were surprisingly high for some raw materials and some participants explained that only a few companies were qualified to produce some materials, which means that they could monopolize the product easily.

The complicated and long approval process was the main reason for the shortages of imported drugs. “Although most of these drugs had substitutes, physicians still preferred to prescribe the imported drugs because of their better effectiveness”, one pharmacist from a tertiary hospital told us. One of the hospital pharmacists noted, “the physicians complained a lot about the shortages of imported drugs.”

Different stakeholders mentioned some other reasons for shortages besides the five most important ones, such as seasonal demands or bad practice in hospital inventory management.

Stakeholders, except for hospital pharmacists, expressed diversified opinions about shortages. Production companies emphasized three other causes: serious delays in payment by hospitals, selective drug use by hospitals, and selective drug distribution by wholesalers. Similarly to producers, wholesalers mentioned serious delays in payment by hospitals and selective drug use by hospitals, and added bad practice in wholesaler inventory management. Local authorities mentioned insufficient information sharing among different parties of the supply chain.

### Solutions for drug shortages

Solving the drug shortage problem requires the participation of different stakeholders, especially the government, because of its complexity. All interviewees provided very good suggestions for the measures against drug shortages. A number of suggestions were made, and the common ones proposed by different stakeholders were extracted for analysis. According to the frequency given by interviewees, the five most important common suggestions were stated in Table 4.

**Table 4:** Solutions for drug shortages proposed by different stakeholders.

Ranking	Solutions
1	Let the market decide the drug price and modify the public bidding mechanism.
2	Establish an information platform for medicines in short supply.
3	Establish a reserve system for medicines in short supply.
4	Enhance the communication among the three parties (manufacturers, wholesalers, and hospitals) in the supply chain.
5	Improve hospital inventory management.

The price issue was the most frequently mentioned problem during the interviews. “All these years, the Chinese National Development and Reform Commission controlled the price for most medicines. The government also compelled price reduction 32 times over the last 20 years. Many drugs vanished every time of the price reduction,” said one hospital representative. Another industry representative complained “The current bidding prices were set a couple of years ago. The prices were too low to manufacture. The government really should readjust the prices according to the changes of the market,”.

Although there was a shortage information-reporting channel provided in the information system of the Drug Procurement Center, its limited functionality made it useless. Most of the interviewees agreed that a “real-time” information platform for medicines in short supply should be established. “Establishing an information platform can be very helpful. If I know some medicines are in shortage in other hospitals, I can prepare well,” one of the hospital pharmacists said. One of the manufacturers said that “if we know some medicines are in widespread shortage and our company also has the qualification and ability to manufacture, we’d like to produce these kinds of medicines.”

Establishing a reserve system for medicines in short supply can reduce the bad effect of the drug shortages. When some drugs were in short supply and no substitutes could be used, public reservation can be beneficial for patients. Several representatives put forward this suggestion with their considerations of the huge cost. One pharmacist said “I seriously doubted that as it cost too much. I don’t think that the government is willing to bear this”. Some of the interviewees proposed to reserve specific medicines for this purpose, such as emergency medicines and clinical essential medicine.

The fourth suggestion appealed for better communication among manufacturers, wholesalers, and hospitals. Because wholesalers were the connecting link between the manufacturers and hospitals, they had greater responsibility for enforcing interaction among the three key parties in the supply chain. A couple of interviewees thought that smoother information exchange among the supply chain parties could reduce the risk of drug shortages and had the effect of early warning.

The last advice was for hospitals. Good inventory management was important for the successful operation of most businesses, so did the hospital management. “Most operators for ordering and inventory management are very young and have just graduated from medical schools. They don’t have any experience or theory in inventory management” one pharmacist told us. One industry representative said, “Their attitude is more important, but the hospitals don’t devote much attention to their inventory management. They usually put the pressure on the suppliers facing shortages.” The pharmacist continued, “We’d like the government to offer seminars to help our operators to improve their inventory management practice.”

Other than these common suggestions, participants also provided some other solutions for the drug shortage problem. Production companies thought that “improving the supervision of raw material manufacturers” and “providing incentives to the manufacturers to produce the drugs in short supply” could be helpful to reduce the risk of drug shortages. Wholesalers proposed that the hospitals should guarantee timely payment.

## Discussion

To the best of our knowledge, this was the first qualitative study to investigate the characteristics, determinants, and possible solutions of drug shortages in China. An inquiry into the reality of drug shortages showed that they were severe in this administrative division. Further, the drivers for drug shortages were complex and multifaceted. The perception of measures to solve these shortages required the involvement of different stakeholders.

As our interviewees reported, the number of drugs in short supply was large and most of them were essential medicines. The introduction of the NEMP aimed to increase the availability of cost-effective medicines and ensure the quality and rational use of these medicines [31].According to the definition defined by the WHO, essential medicines are those that satisfy the priority health care needs of the population, which ought to be available at all times, in the proper dosage forms, to all segments of society [32]. However, the low availability of essential medicines became the most critical point for the implementation of the NEMP and may pose a serious threat to public health in China. The government needed to pay more attention to this problem in the future.

The present study revealed that drug shortages varied at different hospital levels. The shortage of all 87 identified biologicals and chemicals was reported by tertiary hospitals (41.4%) and secondary hospitals (72.4%), which may be caused by the “discrimination” of wholesalers. When the demand for a certain drug exceeded supply in the whole market, wholesalers would satisfy the requirements of tertiary hospitals before that of secondary hospitals. More patients in tertiary hospitals brought them greater negotiating power than secondary hospitals. Wholesalers even provided some medicines with prices below cost to tertiary hospitals to obtain contracts for supplying other medicines.

It seemed that the shortage of antimicrobials, biologicals, and anticancer drugs were more prominent in tertiary hospitals because they reported more shortages. However, it would be inaccurate to say that secondary hospitals do not experience shortages of these medicines. The underlying cause may be that the secondary hospitals did not have as many clinical demands for these medicines as tertiary hospitals, and consequently they will not order these drugs and receive information about their shortage. This also can be recognized as a result of the hierarchical medical system, which requires different levels of medical institutions to treat different diseases.

As our interviewees reported, no single aspect can be indicated as the determinant of drug shortages. However, “too low price” seemed to be a leading complaint. This finding was similar to the “economics” explanation expressed in other literature as the cause of cheap drug shortages in Europe or the US [33–35]. The continuous downwards pressure on generic prices threatened their sustainability [36]. Besides, high price pressure discouraged manufacturers from investing in facilities and quality control, which may cause the “quality issue” that was perceived as another big problem of cheap drug shortages in Europe and the US. In China, the “quality issue” was currently not a main reason for the drug shortages. The compulsory price-reduction policy and public bidding put high pressure on drug prices, especially for those drugs with low technical barriers. A large number of manufacturers can produce the product and participate in the tender, which made the public bidding more competitive and the bidding price much lower. On one side, manufacturers had less incentive to produce these low-price drugs as it brought less profit than producing other expensive ones. On the other side, the hospitals had financial incentives to prescribe expensive drugs over low-price drugs because they were allowed to earn a 15% mark-up on the sale of pharmaceutical products[37]. As the low-price drugs were more susceptible to shortages, some provincial governments began to adopt a new procurement procedure for those “low-price drugs.” Under this new procurement mechanism, all the public hospitals can order from those manufacturers as long as they met the technical and quality requirements (in the previous procedure, only several bid winners can be the purchase sources) [38]. Too low market demand was also claimed to be a major cause of emergency and orphan drug shortages in China. This factor has been discussed rarely in other countries, but has been much considered in China [39–41]. The root of this problem may still come down to the “low price” issue. Most emergency medicines were on the list of low-price drugs, within the scope of the National Development and Reform Commission Pricing List released in April 2014 [42]. For example, the tendering price of pralidoxime iodide in Shaanxi Province was 0.2236$ (1$ = 6.3865RMB) per ampoule (0.5 g: 20 mL), which may leave manufacturers little or no profit. Although the prices of most orphan drugs were practically high, the manufacturers still cannot make profits because of the high cost of production.

According to our study, drug shortages may also be impacted by the GMP and materials issues. These two factors were similar to the “manufacturing difficulties” and “raw and bulk material unavailability” described in other literature [9,15, 43–45]. Drug shortages may occur when the manufacturer cannot comply with the current GMP and is forced to halt production, especially when the manufacturer was the primary or sole producer of the drug. In the US, manufacturer problems, such as antiquated manufacturing equipment and insufficient investment, was usually the cause of this compliance problem. However, in China, manufacturers complained that the long process of renewing authentication was the main problem. The fundamental reason based on materials issues also differed between China and the US. In China, material issues included deficiencies of raw materials and the monopoly behavior of the raw material manufacturers. In the US, this problem was mainly caused by the susceptibility of the global supply chain because 80% of the raw materials came from other countries [9].

After identifying all of these causes of drug shortages, a perfect strategy to solve this problem was to find a corresponding solution for each cause. However, some solutions may be not feasible or not a priority at least in this stage of the Chinese health system. For the last three common causes in Table 3, the stakeholders could not reach an agreement on the solutions. For example, although all stakeholders recognized that the “material issues” was a reason for some drug shortages, most interviewees thought that it would be difficult to implement countermeasures. In their opinion, importing raw materials may not help because companies in the US, which imported 80% of their raw materials, face the same problem. Regulation of raw material manufacturers to prevent unreasonable increase in materials prices would be a great challenge for the current Chinese health system.

Despite all the difficulties, some significant suggestions were proposed by different stakeholders. The government needed to play a leading role in the management of drug shortages. First, for the “too low prices” and “too low market demands,” the practical solution would be to “let the market decide the drug price.” For these two kinds of medicines, strict price control may easily lead to them vanishing from the market as the manufacturers were very difficult to obtain profits. When the price was decided by the market, drug price may rise.“A higher price but available in the market” benefited patients more than “a lower price but no access”. Moreover, the drug price would not increase too much because of the market competition. Besides, government can monitor the price to make sure they remain stable as what the Chinese government did right now. Second, although respondents indicated that information about drug shortages was communicated between the different parties in the supply chain, the information was not provided in advance. Therefore, establishing an information platform where public hospitals can share information about drug shortages will increase collaboration between hospitals. Furthermore, if the wholesalers and manufacturers became involved in reporting information, healthcare workers can anticipate a drug shortage at an early stage. Consequently, the effects of drug shortages can be reduced. Many researchers recommended this strategy. The U.S. FDA even required all manufacturers to inform them of any impending shortages so they could prevent and mitigate the problem [46]. Third, reserving an amount of medicines that were frequently in shortage, especially for emergency medicines, would greatly reduce the clinical effects of drug shortages. However, because reserving medicines required the investment of a great deal of money, supply chain parties should be conscientious in choosing medicines and deciding the quantity of the reserve. Wholesalers also played an important role in managing drug shortages. If they can be more active in promoting communication between the manufacturers and the hospitals, information can be shared in the supply chain more easily and most drug shortages can be avoided. Hospitals also needed to raise their awareness of good inventory management, which can not only decrease drug shortages in hospitals, but also reduce their inventory costs and improve their competitiveness [9].

## Limitations

The small number of interviewees was a limitation of this study. However, we interviewed the high-level managers or officers who were most familiar with the drug shortages in each organization, which guaranteed that our information was accurate and representative. Although we had concerns that some of the interviewees would be afraid to speak frankly, our impression was that they all felt comfortable and expressed themselves freely, and the results reflected that. Furthermore, participants were Shaanxi Province institutions; therefore, our results were representative only for western China.

## Conclusions

Reports of drug shortages have been increasing in western China. Essential medicines were the most frequently reported drugs in shortage, and secondary hospitals encountered more drug shortages than did tertiary hospitals. Antimicrobials, biologicals, and anticancer drugs were the most common drugs in short supply in tertiary hospitals while cardiovascular drugs, hormones, and endocrine system agents were usually on the list of short supply in secondary hospitals. Five main determinants of drug shortages were identified throughout the present study: too low prices, too low market demands, GMP issues, materials issues, and approval issues with imported drugs. “Too low prices” was currently the key reason for most drug shortages in China. To solve this problem, all stakeholders, especially the government, needed to participate in managing the drug shortages. Participants proposed the establishment of “an information platform for medicines in short supply” among four other common suggestions.

Further research was needed to quantify and identify the underlying causes and actual impact of drug shortages in China and develop long-term prevention strategies.

## Supporting Information

S1 File Interview guide—set of questions for manufacturers. (PDF)

S2 File Interview guide—set of questions for wholesalers. (PDF)

S3 File Interview guide—set of questions for hospital pharmacists. (PDF)

S4 File Interview guide—set of questions for local authorities. (PDF)

S5 File Permission and Information Sheet. (PDF)

## References

[1] Gray A, Manasse HR. Shortages of medicines: a complex global challenge. Bulletin of World Health Organization. 2012; 90(3): 158–158A. 10.2471/BLT.11.101303 PMC3314203 22461706

[2] Beerten E, Bonheure F. Autour du monde-des indisponsibilités de medicaments. Annales Pharmaceutiques Belges. 2011; 15: 11–14.

[3] Kaakeh R, Sweet BV, Reilly C, Bush C, Deloach S, Higgins B, et al Impact of drug shortages on US health systems. American Journal of Health-System Pharmacy. 2011; 68(19): 1811–1819. 10.2146/ajhp110210 21930639

[4] Casassus B. Europe urged to take action on drug shortages. The Lancet. 2015; 385(9975): 1279–1280. 10.1016/S0140-6736(15)60667-5 25890899

[5] Kaiser J. Shortages of cancer drugs put patients, trials at risk. Science. 2011; 332(6029): 523 10.1126/science.332.6029.523 21527686

[6] Rosoff PM. Unpredictable drug shortages: an ethical framework for short-term rationing in hospitals. American Journal of Bioethics. 2012; 12(1): 1–9. 10.1080/15265161.2011.634483 22220948

[7] Kux L. Center for Drug Evaluation and Research, Approach to Addressing Drug Shortage; Public Workshop; Request for Comments. Federal Register, 2011.

[8] Baumer AM, Clark AM, Witmer DR, Geize SB, Vermeulen LC, Deffenbaugh JH. National survey of the impact of drug shortages in acute care hospitals. American Journal of Health-System Pharmacy. 2004; 61(19): 2015–2022. 15509124 10.1093/ajhp/61.19.2015

[9] Fox ER, Birt A, James KB, Kokko H, Salverson S, Soflin DL. ASHP Guidelines on Managing Drug Product Shortages in Hospitals and Health Systems. American Journal of Health-System Pharmacy. 2009; 66(15):1399–1406. 10.2146/ajhp090026 19635779

[10] U.S. Food and Drug Administration (FDA). A review of FDA’s approach to medical product shortages US Department of Health and Human Services, 2011.

[11] Haninger K, Jessup A, Koehler K. Economic analysis of the causes of drug shortages. Department of Health and Human Services, 2011.

[12] Schweitzer SO. How the US Food and Drug Administration can solve the prescription drug shortage problem. American Journal of Public Health. 2013; 103(5): e10–e14. 10.2105/AJPH.2013.301239 23488502 PMC3698821

[13] Kweder SL, Dill S. Drug shortages: the cycle of quantity and quality. Clinical Pharmacology & Therapeutics. 2013; 93(3): 245–251. 23340474 10.1038/clpt.2012.235

[14] Generics and Biosimilar Initiative. UK drug shortages are far from being solved. 13 Jan 2012. Available: http://gabionline.net/layout/set/print/content/view/full/1635 Accessed 1 April 2016.

[15] Pauwels K, Simoens S, Casteels M, Huys I. Insights into European Drug Shortages: A Survey of Hospital Pharmacists. PLOS ONE. 2015; 10(3): e0119322 10.1371/journal.pone.0119322 25775406 PMC4361582

[16] Bogaert P, Bochenek T, Prokop A, Pilc A. A Qualitative Approach to a Better Understanding of the Problems Underlying Drug Shortages, as Viewed from Belgian, French and the European Union’s Perspectives. PLOS ONE. 2015; 10(5): e0125691 10.1371/journal.pone.0125691 25942432 PMC4420462

[17] Canadian Medical Association (CMA). ePanel Survey Summary: Generic prescription drug shortages. 2011. Available: http://www.cma.ca/advocacy/generic-prescription-drug-shortages Accessed 1 April 2016.

[18] Canadian Pharmacists Association (CPA). Canadian drug shortages survey: final report Ottawa: Canadian Pharmacists Association 12 2010 Available: http://www.pharmacists.ca/cpha-ca/assets/File/cpha-on-the-issues/DrugShortagesReport.pdf Accessed 1 April 2016.

[19] Kaposy C. Drugs, Money, and Power: The Canadian Drug Shortage. Journal of Bioethical Inquiry. 2014; 11(1):1–5. 24357073 10.1007/s11673-013-9494-z

[20] Wang YG. Drug shortages are intensified under price ceiling. Decision and Information: financial observation. 2005; 12: 36–37. (in Chinese)

[21] Liu B. Economic Analysis on the Shortage of Low-price Essential Medicines Caused by Price-lowering Policy. China Pharmacy. 2007; 18: 2481–2483. (in Chinese)

[22] Fang Y, Wagner AK, Yang S, Jiang M, Zhang F, Ross-Degnan D. Access to affordable medicines after health reform: evidence from two cross-sectional surveys in Shaanxi Province, western China. Lancet Global Health, 2013, 1(4):e227–e237. 10.1016/S2214-109X(13)70072-X 25104348

[23] Liu M, Wu Q. Study on the shortage of essential drugs in China. China Pharmacy 2012; 23: 673–674. (in Chinese)

[24] Zhang Y, Tang S. Supply and security policies of cheap commonly used drugs based on supply chain theory. Chinese General Practice 2015; 18: 243–247. (in Chinese)

[25] Ding Y. National Health and Family Planning Commission and the State Administration of Traditional Chinese Medicine of the P.R.C ensure drug supply. Journal of Traditional Chinese Medicine Management 2015; 23: 99. (in Chinese)

[26] Susie D, Jin A. Drug shortages in developed countries-reasons, therapeutic consequences, and handling. European Journal of Clinical Pharmacology 2014; 70: 1405–1412. 10.1007/s00228-014-1747-1 25228250

[27] Wikipedia. Shaanxi. 1 April 2016. Available: http://en.wikipedia.org/wiki/Shaanxi. Accessed 1 April 2016.

[28] WHO. The western area health initiative. 2012. Available: http://www.wpro.who.int/china/areas/western_area_health_initiative/en/ Accessed 1 April 2016.

[29] Zeng W, Zhen J, Feng M, Campbell SM, Finlayson AE, Godman B. Analysis of the influence of recent reforms in China: cardiovascular and cerebrovascular medicines as a case history to provide future direction. Journal of comparative effectiveness research 2014; 3(4): 371–386. 10.2217/cer.14.28 25275234

[30] National Health and Family Planning Commission of the People’s Republic of China. National essential medicine list. 13 Mar 2013. Available: http://www.moh.gov.cn/mohywzc/s3580/201303/f01fcc9623284509953620abc2ab189e/files/961cfc3a86584f8888e9140b1c208438.pdf Accessed 1 April 2016.

[31] WHO. How to Develop and Implement a National Drug Policy 2nd edition Malta: World Health Organization, 2001a.

[32] WHO. WHO Medicines Strategy. Revised procedure for Updating WHO’s Model List of Essential Drugs. Geneva: WHO Technical Report Series, 2001b.

[33] Chabner BA. Drug shortages-a critical challenge for the generic-drug market. New England Journal of Medicine. 2011; 365(23):2147–2149. 10.1056/NEJMp1112633 22040167

[34] Pauwels K, Huys I, Casteels M, Simoens S. Drug shortages in European countries: a trade-off between market attractiveness and cost containment? BMC Health Services Research. 2014; 14(1): 1–9. 25257912 10.1186/1472-6963-14-438 PMC4263120

[35] Woodcock J, Wosinska M. Economic and technological drivers of generic sterile injectable drug shortages. Clinical Pharmacology & Therapeutics. 2013; 93(2): 170–176. 23337525 10.1038/clpt.2012.220

[36] Dylst P, Vulto A, Godman B, Simoens S. Generic medicines: solutions for a sustainable drug market? Applied health economics and health policy 2013; 11(5):437–443. 10.1007/s40258-013-0043-z 23846572

[37] Meng Q, Cheng G, Silver L, Sun X, Rehnberg C, Tomson G. The impact of China's retail drug price control policy on hospital expenditures: a case study in two Shandong hospitals. Health Policy & Planning 2005; 20(3):185–196. 15840634 10.1093/heapol/czi018

[38] Fang LB, Zuo GY, Jia LY. The Comparative Study on Low-cost Drugs Online Procurement Policy in China. Chinese Health Service Management. 2015; 32: 610–612. (in Chinese)

[39] Gong SW, Zhang L, Jin S, Li LL. Management strategy for raising the accessibility to orphan drugs in China. Chinese Journal of Hospital Administration. 2010; 26: 126–130. (in Chinese)

[40] Gu JL, Lu YQ, Zhang R, Yue Y, Duan Yx, Xu Lz. Research on rare disease drugs attainability strategy in China. Soft Science of Health. 2013; 27: 325–327. (in Chinese)

[41] Liu XK, Dong NQ, Yan YJ. The reasons, impact and measures for the shortage of clinical cheap drugs. Journal of North Pharmacy. 2013; 10: 88–89. (in Chinese)

[42] National Development and Reform Commission. (NDRC). A notice about improving the management of low price medicines. 26 April 2014. Available: http://www.sdpc.gov.cn/zcfb/zcfbtz/201405/t20140508_610853.html Accessed 1 April 2016. (in Chinese)

[43] Butler D. Iran hit by drug shortage. Nature. 2013; 504(7478):15–16. 10.1038/504015a 24305129

[44] Ventola CL. The drug shortage crisis in the United States: causes, impact, and management strategies. Arquivos Brasileiros De Endocrinologia E Metabologia. 2011; 36(11): 740–57. PMC3278171 22346307

[45] Printz C. Medication shortages threaten cancer care. Cancer. 2012; 118(2): 289–291. 10.1002/cncr.27386 22222939

[46] Tucker ME. US drug shortages: a disappearing problem? British Medical Journal. 2012; 345: 900–902. 10.1136/bmj.e8551 23261759

