# Supplementary material for: Current Situation, Determinants, and Solutions to Drug Shortages in Shaanxi Province, China: A Qualitative Study
Source: PLoS One. 2016 Oct 25;11(10):e0165183. doi: 10.1371/journal.pone.0165183 (PMC5079602; doi:10.1371/journal.pone.0165183)
Supplement: S1 File — (PDF) [file pone.0165183.s001.pdf]

SUPPLEMENTARY MATERIAL

TEXT S1. INTERVIEW GUIDE –MANUFACTURERS

In this context, we define drug shortages as a deficiency in the supply of a medicinal product which hinders meeting the demand of the product at a wholesaler level.

General information

1. Name of the medicinal producers: _______________
2. Gender: ____________________
3. Age: _________________________
4. Years of medicine related working experience : _________________

Present situation of drug shortage

1. During the past 12 months, has your company ever met the situation in which your medicinal products cannot meet the demands of downstream wholesalers?

   ☐Yes ☐NO ☐unclear

   If yes, please list several names of those drugs and corresponding reasons.

   ____

   ____

2. According to your experience, what is the trend of the number of drugs in shortage during the past 12 months?

   ☐increase strongly ☐increase ☐keep constant

   ☐decrease ☐decrease strongly ☐unclear

3. Has your company ever sent out notifications regarding to drug shortages?

   ☐never ☐hardly ever ☐occasionally ☐often ☐always

   If yes, whom does your company often give the notification to ?

   ☐wholesalers ☐healthcare institutions

   ☐government authority ☐others

4. Has your company ever taken some measures to prevent drug shortages?

   ☐Yes ☐NO ☐unclear

   If yes, please explain the details of the measures:_________________________

   ____

5. Does your company have specific rules or procedures to struggle with drug shortages?

   ☐Yes ☐NO ☐Unclear

   If yes, please explain the details of the rules or procedures:______________________

   ____

6. Do you think that your company is affected by drug shortages?

   ☐Yes ☐NO ☐Unclear

   If yes, please explain the details of effects:____________________________________

   ____

The causes of drug shortages

What are the reasons for drug shortages in your opinion?

1. **What are the determinants leading to drug shortages from the perspectives of medicine manufacturers?**
   - ✓ Raw materials
   - ✓ Change of GMP request
   - ✓ Internal decision-making process
   - ✓ Others……
2. **What are the determinants leading to drug shortages from the perspectives of drug wholesalers?**
   - ✓ The scales and number of wholesalers
   - ✓ Distribute the medicines selectively
   - ✓ Others……
3. **What are the determinants leading to drug shortages from the perspectives of healthcare institutions?**
   - ✓ Poor drug inventory management
   - ✓ Poor communication with wholesalers
   - ✓ Use some drugs selectively
   - ✓ Others……
4. **What are the determinants leading to drug shortages from the perspectives of the characteristics of medicines?**

- ✓ Price
- ✓ Demand
- ✓ Manufacturing process
- ✓ Others……

5. **Are there any other reasons causing drug shortages?**
6. **Among those causes above mentioned, what are the three most important ones in your opinion?**

Solutions for drug shortages

How to solve the drug shortage problem in your opinion?

1. **What the governmental authorities could do to manage drug shortage problem?**
    - ✓ Strengthen the supervision of material market
    - ✓ Strengthen the management of drug registration
    - ✓ Modify the drug pricing policy
    - ✓ Establish the platform for managing drug shortages
    - ✓ Build drug shortages related laws and regulations
    - ✓ Establish pharmaceutical reserve system for drugs in short supply
    - ✓ Establish the guideline managing drug shortages
    - ✓ Strengthen the behaviours of agents of imported drugs
    - ✓ Motivate the drug manufactures producing drugs in short supply
    - ✓ Set up one specific department to deal with the problem?
    - ✓ Others……
2. **What the drug manufacturers could do to deal with drug shortages?**
    - ✓ Active in promoting the drug quality and meet GMP request
    - ✓ Making “preventing drug shortage” as one of the business goals
    - ✓ Establish good communications with suppliers of raw materials
    - ✓ Improve the prediction of market demand
    - ✓ Enhance the flexibility to meet the demand uncertainty in the market
    - ✓ Build contingency mechanism for drug shortages problem
    - ✓ Improve the supply of the alternatives of drugs in short supply
    - ✓ Others……

3. **What the wholesalers could do to deal with drug shortages?**
   - ✓ Standardize the distribution behaviour in the enterprises
   - ✓ Enhance the communication with manufactures and other pharmaceutical agents
   - ✓ Others……
4. **What the healthcare institutions could do to deal with drug shortages?**
   - ✓ Increase the number of wholesalers
   - ✓ Improve the inventory management
   - ✓ Reinforce the management of wholesalers
   - ✓ Establish the guidelines for managing drug shortages and put into action
   - ✓ Others……
5. **Are there any other solutions to manage this problem?**
6. **Among those solutions, what are the three most important ones in your opinion?**
