# Supplementary material for: Current Situation, Determinants, and Solutions to Drug Shortages in Shaanxi Province, China: A Qualitative Study
Source: PLoS One. 2016 Oct 25;11(10):e0165183. doi: 10.1371/journal.pone.0165183 (PMC5079602; doi:10.1371/journal.pone.0165183)
Supplement: S2 File — (PDF) [file pone.0165183.s002.pdf]

SUPPLEMENTARY MATERIAL

TEXT S2. INTERVIEW GUIDE –WHOLESALERS

In this context, we define drug shortages as a deficiency in the supply of a medicinal product which hinders meeting the demand of the product at a hospital level.

General information

1. Name of the wholesale enterprise: ________________
2. Gender: _____________________
3. Age: __________________________
4. Years of medicine related working experience : __________________

Present situation of drug shortage

1. During the past 12 months, has your company ever met the situation in which the medicinal products you operated cannot meet the demands?

   ☐Yes ☐NO ☐unclear

   If yes, please list several names of those drugs and corresponding reasons.

   ____

   ____

2. According to your experience, what is the trend of the number of drugs in shortage within your company during the past 12 months?

   ☐increase strongly ☐increase ☐keep constant

   ☐decrease ☐decrease strongly ☐unclear

3. Has your company ever received notifications regarding to drug shortages?

   ☐never ☐hardly ever ☐occasionally ☐often ☐always

   If yes, from whom does your company receive the notification?

   ☐manufactures ☐healthcare institutions

   ☐government authority ☐others

4. Has your company ever sent out notifications regarding to drug shortages?

   ☐never ☐hardly ever ☐occasionally ☐often ☐always

   If yes, whom does your company send the notification to?

   ☐healthcare institutions ☐government authority ☐others

5. Have your company ever taken some measures to prevent drug shortages?

☐Yes ☐NO ☐unclear

If yes, please explain the details of the measures:___________________________

____

6. Does your company have specific rules or procedures to struggle with drug shortages?

☐Yes ☐NO ☐unclear

If yes, please explain the details of the rules or procedures:______________________

____

7. Do you think that your company is affected by drug shortages?

☐Yes ☐NO ☐unclear

If yes, please explain the details of effects:____________________________________

____

The causes of drug shortages

What are the reasons for drug shortages in your opinion?

1. **What are the determinants leading to drug shortages from the perspectives of medicine manufacturers?**
   - ✓ Raw materials
   - ✓ Change of GMP request
   - ✓ Internal decision-making process
   - ✓ Others……
2. **What are the determinants leading to drug shortages from the perspectives of drug wholesalers?**
   - ✓ The scales and number of wholesalers
   - ✓ Distribute the medicines selectively
   - ✓ Others……
3. **What are the determinants leading to drug shortages from the perspectives of healthcare institutions?**
   - ✓ Poor drug inventory management
   - ✓ Poor communication with wholesalers

- ✓ Use some drugs selectively
- ✓ Others……

4. **What are the determinants leading to drug shortages from the perspectives of the characteristics of medicines?**
    - ✓ Price
    - ✓ Demand
    - ✓ Manufacturing process
    - ✓ Others……
5. **Are there any other reasons causing drug shortages?**
6. **Among those causes above mentioned, what are the three most important ones in your opinion?**

Solutions for drug shortages

How to solve the drug shortage problem in your opinion?

1. **What the governmental authorities could do to manage drug shortage problem?**
    - ✓ Strengthen the supervision of material market
    - ✓ Strengthen the management of drug registration
    - ✓ Modify the drug pricing policy
    - ✓ Establish the platform for managing drug shortages
    - ✓ Build drug shortages related laws and regulations
    - ✓ Establish pharmaceutical reserve system for drugs in short supply
    - ✓ Establish the guideline managing drug shortages
    - ✓ Strengthen the behaviours of agents of imported drugs
    - ✓ Motivate the drug manufactures producing drugs in short supply
    - ✓ Set up one specific department to deal with the problem?
    - ✓ Others……
2. **What the drug manufacturers could do to deal with drug shortages?**
    - ✓ Active in promoting the drug quality and meet GMP request
    - ✓ Making "preventing drug shortage" as one of the business goals
    - ✓ Establish good communications with suppliers of raw materials
    - ✓ Improve the prediction of market demand

- ✓ Enhance the flexibility to meet the demand uncertainty in the market
- ✓ Build contingency mechanism for drug shortages problem
- ✓ Improve the supply of the alternatives of drugs in short supply
- ✓ Others……

3. **What the wholesalers could do to deal with drug shortages?**
   - ✓ Standardize the distribution behaviour in the enterprises
   - ✓ Enhance the communication with manufactures and other pharmaceutical agents
   - ✓ Others……
4. **What the healthcare institutions could do to deal with drug shortages?**
   - ✓ Increase the number of wholesalers
   - ✓ Improve the inventory management
   - ✓ Reinforce the management of wholesalers
   - ✓ Establish the guidelines for managing drug shortages and put into action
   - ✓ Others……
5. **Are there any other solutions to manage this problem?**
6. **Among those solutions, what are the three most important ones in your opinion?**
