# Supplementary material for: Current Situation, Determinants, and Solutions to Drug Shortages in Shaanxi Province, China: A Qualitative Study
Source: PLoS One. 2016 Oct 25;11(10):e0165183. doi: 10.1371/journal.pone.0165183 (PMC5079602; doi:10.1371/journal.pone.0165183)
Supplement: S3 File — (PDF) [file pone.0165183.s003.pdf]

SUPPLEMENTARY MATERIAL

TEXT S3. INTERVIEW GUIDE –HOSPITAL PHARMACISTS

In this context, we will define drug shortages as a deficiency in the supply of a medicinal product which hinders meeting the demand of the product at a patient level.

General information

1. Name of the hospital: ________________
2. Gender: ______________________
3. Age: ___________________________
4. Years of medicine related work experience: ___________________

List specific names of drugs in shortage (generic name, brand name, strength and dosage form) during last 12 months in your hospital.

Data collection form

Number	Generic name	Brand name	Strength	Dosage form
1				
2				
3				
4				
5				
6				
7				
8				
9				
10				

The causes of drug shortages

What are the reasons for drug shortages phenomena in your opinion?

1. **What are the determinants leading to drug shortages from the perspectives of medicinal producers?**
   - ✓ Raw materials
   - ✓ change of GMP request
   - ✓ few producers with the license to produce corresponding drugs
   - ✓ internal decision-making process

2. **What are the determinants leading to drug shortages from the perspectives of drug wholesalers?**
   - ✓ The scales and number of wholesalers
   - ✓ Distribute the medicines selectively
3. **What are the determinants leading to drug shortages from the perspectives of healthcare institutions?**
   - ✓ Poor drug inventory management
   - ✓ Poor communication with wholesalers
   - ✓ Use drugs selectively
4. **What are the determinants leading to drug shortages from the perspectives of medicines themselves?**
   - ✓ Price
   - ✓ Low demands or uncertainty demands
   - ✓ Complex manufacture processes
5. Are there any other reasons causing drug shortages?
6. Among those causes, what are the three most important ones in your opinion?

Solutions for drug shortages

How to solve the drug shortage problem in your opinion?

1. **What the governmental authorities could do to manage drug shortage problem?**
   - ✓ Strengthen the supervision of material market
   - ✓ Strengthen the management of drug registration
   - ✓ Modify the drug pricing policy
   - ✓ Establish the platform for managing drug shortages
   - ✓ Set up dedicated department to deal with drug shortages
   - ✓ Build drug shortages related laws and regulations
   - ✓ Establish pharmaceutical reserve system for drugs in short supply
   - ✓ Establish the guideline managing drug shortages
   - ✓ Others……
2. **What the drug manufactures could do to deal with drug shortages?**
   - ✓ Active in promoting the drug quality and meet the GMP request

- ✓ Take “preventing drug shortage” as one of the business goals
- ✓ Establish good communications with suppliers of raw materials
- ✓ Improve the prediction of market demands
- ✓ Enhance production flexibility to meet the demand uncertainty in the market
- ✓ Build contingency mechanism for drug shortages problem
- ✓ Improve the supply of the alternatives of drugs in short supply
- ✓ Others……

3. **What the wholesalers could do to deal with drug shortages?**
   - ✓ Standardize the distribution behaviour in the enterprises
   - ✓ Enhance the communication with manufactures and other pharmaceutical agents
   - ✓ Others……
4. **What the healthcare institutions could do deal with drug shortages?**
   - ✓ Increase the number of wholesalers
   - ✓ Improve the inventory management
   - ✓ Reinforce the management of wholesalers
   - ✓ Establish the guidelines for managing drug shortages and put into action
5. **Are there any other solutions to manage this problem?**
6. **Among those solutions, what are the three most important ones in your opinion?**
