# Supplementary material for: Current Situation, Determinants, and Solutions to Drug Shortages in Shaanxi Province, China: A Qualitative Study
Source: PLoS One. 2016 Oct 25;11(10):e0165183. doi: 10.1371/journal.pone.0165183 (PMC5079602; doi:10.1371/journal.pone.0165183)
Supplement: S5 File — (PDF) [file pone.0165183.s005.pdf]

Permission and Information Sheet

Study to Investigate Current situation, determinants, and solutions to drug shortages in Shaanxi Province, China

Interviewer: ____

Purpose of the study: The purpose of this study is to analyze, characterize, and assess the drug shortages, and identify possible solutions in Shaanxi Province, western China.
It means to have in-depth understandings of drug shortage problems from the perspective of different stakeholders.

Methodology: Semi-structured interviews were performed to gather information about drug shortages from representatives of hospital pharmacists, wholesalers, pharmaceutical producers, and local health authorities.

Confidentiality: The interview will be audio-recorded. The information gathered from you will remain confidential and only the researchers will have access to it. Your name will not be used anywhere in the study. Data gathered from this study will be kept in a safe cabinet.

Before agreeing to this study, it is important that you have clearly understood the purpose of the study. This agreement states that you have understood everything about the study and that you are giving us permission to use information gathered from you for the study.

Date: _____________
